# Supplementary material for: Myroides species, pathogenic spectrum and clinical microbiology sight in Mexican isolates
Source: PLoS One. 2024 Nov 4;19(11):e0310262. doi: 10.1371/journal.pone.0310262 (PMC11534234; doi:10.1371/journal.pone.0310262)
Supplement: S1 Fig — (DOCX) [file pone.0310262.s001.docx]

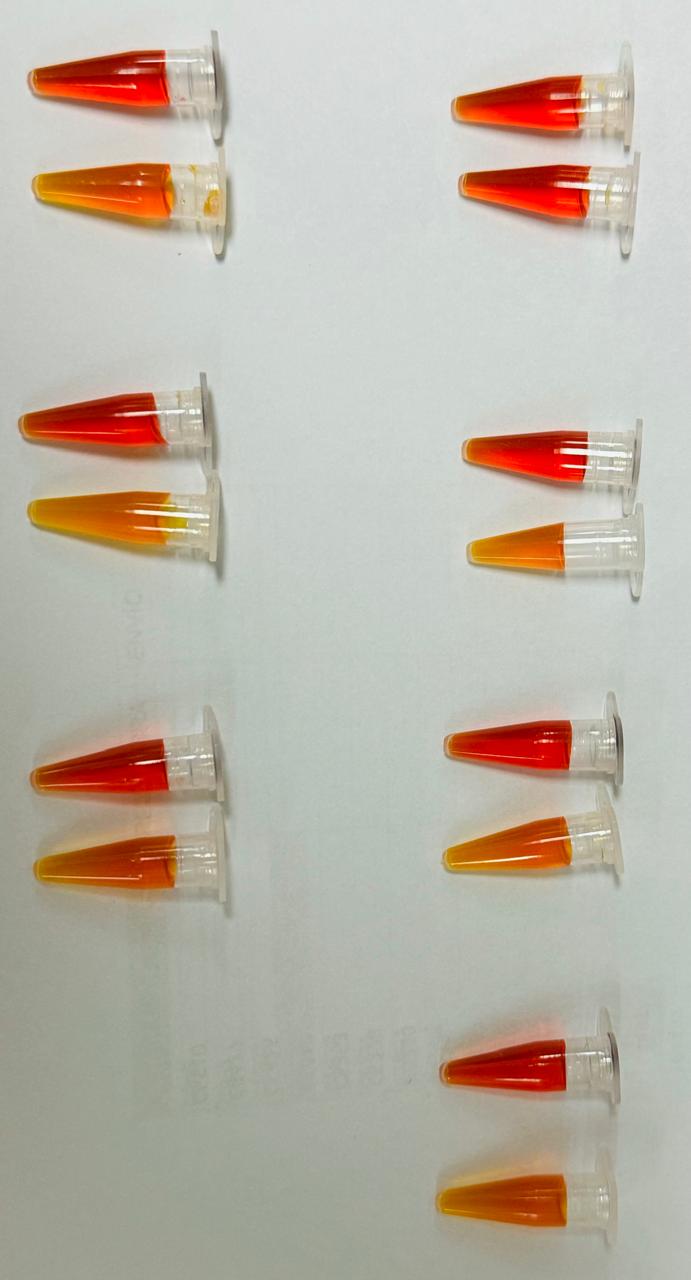


C2723

Negative

EB1487

Positive

C1519

Positive

C4411

Positive

C1996

Positive

C4256

Positive

C4067

Positive

Supplementary Fig 1. CARBA NP in *Myroides spp.* Clinical strains.
