# Supplementary material for: Myroides species, pathogenic spectrum and clinical microbiology sight in Mexican isolates
Source: PLoS One. 2024 Nov 4;19(11):e0310262. doi: 10.1371/journal.pone.0310262 (PMC11534234; doi:10.1371/journal.pone.0310262)
Supplement: S2 Fig — MWM: Molecular weight marker. (DOCX) [file pone.0310262.s002.docx]

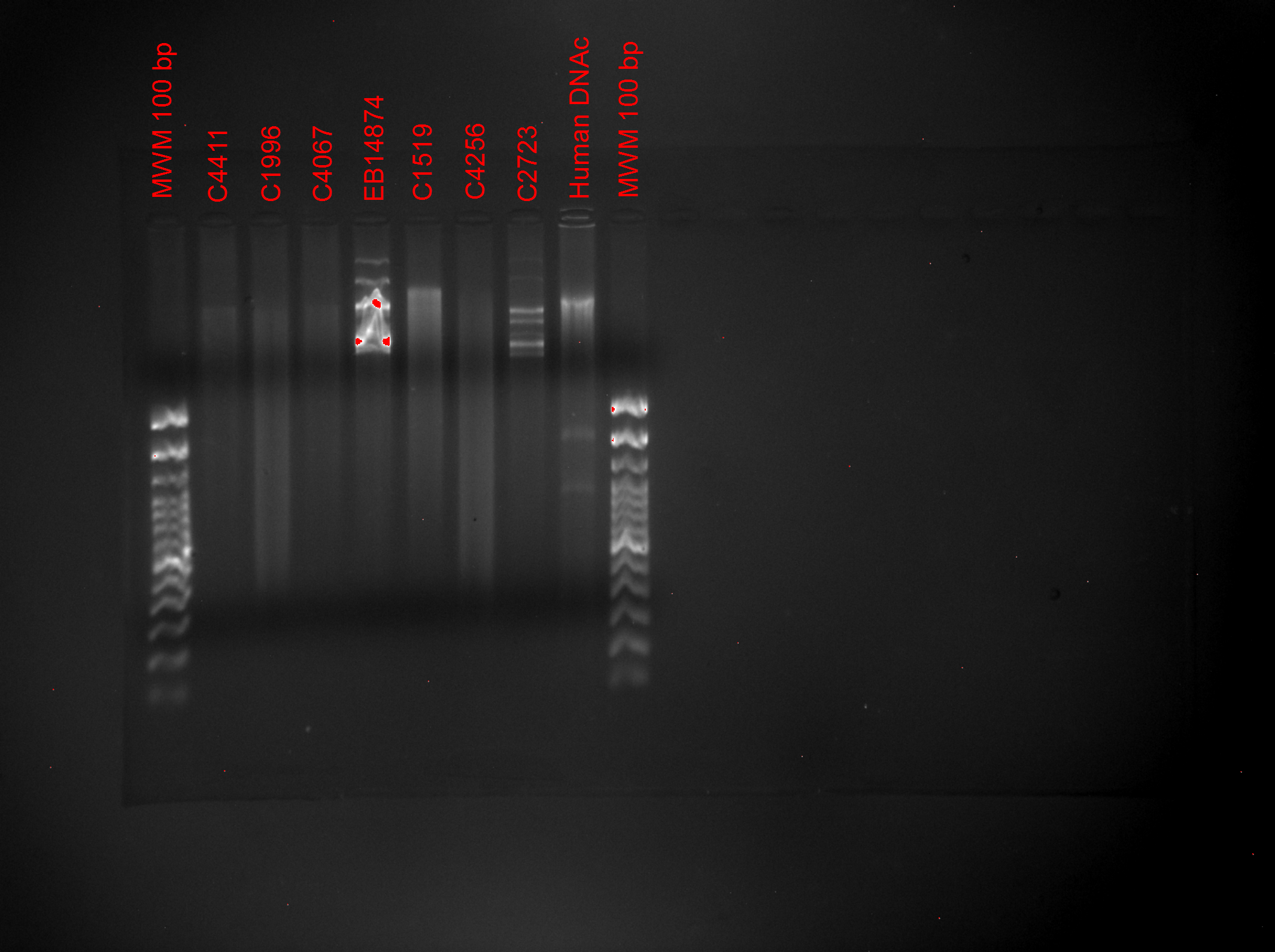


Fig 2 Supplementary. Plasmids extracted from *Myroides spp.* Clinical strains.

MWM: Molecular weight marker.
