# Supplementary material for: Myroides species, pathogenic spectrum and clinical microbiology sight in Mexican isolates
Source: PLoS One. 2024 Nov 4;19(11):e0310262. doi: 10.1371/journal.pone.0310262 (PMC11534234; doi:10.1371/journal.pone.0310262)
Supplement: Attachment — Submitted filename: PONE-D-24-22192 reviewed_olorukooba.pdf [file pone.0310262.s004.pdf]

PLOS ONE

Myroides species, pathogenic spectrum and clinical microbiology sight in Mexican isolates.

--Manuscript Draft--

**Manuscript Number:**	PONE-D-24-22192
**Article Type:**	Research Article
**Full Title:**	Myroides species, pathogenic spectrum and clinical microbiology sight in Mexican isolates.
**Short Title:**	Infections related to Myroides spp.
**Corresponding Author:**	Luis Esau Lopez Jacome, PhD<br>Instituto Nacional de Rehabilitación: Instituto Nacional de Rehabilitacion Luis Guillermo Ibarra Ibarra<br>Ciudad de México, MEXICO
**Keywords:**	Myroides spp.; Antibiotic resistance; infection; skin and soft tissue; pathogen
**Abstract:**	Myroides is a bacterial genus of opportunistic bacteria responsible for diverse infections including in the skin and soft tissues, urinary tract, cardiovascular system, and bacteremia, although the incidence of his reported infections is low, it is increasing, likely due the use of better bacterial identification methods, but also perhaps due an increase in its prevalence.<br><br>Objective<br><br>To describe the microbiological and genetic characteristics of seven different Myroides spp. clinical strains and comment on their phylum, pathogenic and resistance characteristics.<br><br>Methods<br><br>Myroides spp., strains were identified by miniaturized biochemistry and MALDI-ToF. Susceptibility tests were performed according to CLSI recommendations by broth microdilution. Whole genome sequencing was performed for each strain and bioinformatics analysis were performed.<br><br>Results<br><br>Strains were identified at genus level by two methodologies. Our results revealed that likely four strains belong to the species Myroides odoratimimus, while the other two may be undescribed ones. Remarkably, all isolates harbored several genes encoding antibiotic resistance determinants for ß-lactams, aminoglycosides and glycopeptides and in concordance, presented high levels of resistance, against these antibiotics; moreover, the presences of carbapenemases were evidenced by meropenem degrading activity in five isolates and at least two strains possessed plasmids harboring mainly ribosomal RNA genes, tRNAs and genes encoding proteins with unknown functions.<br><br>Conclusions<br><br>Our study increases the knowledge about the biology of this understudied genus and highlights the potential of Myroides to emerge as a broader cause of recalcitrant opportunistic infections.
**Order of Authors:**	Claudia Adriana Colin Castro, PhD
	Jossue Mizael Ortiz Álvarez, PhD
	Cindy Fabiola Hernández Pérez, MsC
	Melissa Hernández Durán, MsC
	María de Lourdes García Hernández, MSc

Powered by Editorial Manager® and ProduXion Manager® from Aries Systems Corporation

	María Guadalupe Martínez Zavaleta, BsC
	Noé Becerra Lobato, MSc
	Mercedes Isabel Cervantes Hernández, BSc
	Graciela Rosas Alquicira, BSc
	Guillermo Cerón González
	Braulio Josué Méndez Sotelo, MSc
	Rodolfo García Contreras, PhD
	Rafael Franco Cendejas, MSc
	Luis Esau Lopez Jacome, PhD
**Additional Information:**	
**Question**	**Response**
**Financial Disclosure**<br><br>Enter a financial disclosure statement that describes the sources of funding for the work included in this submission. Review the submission guidelines for detailed requirements. View published research articles from *PLOS ONE* for specific examples.<br><br>This statement is required for submission and **will appear in the published article** if the submission is accepted. Please make sure it is accurate.<br><br>**Funded studies**<br>Enter a statement with the following details:<br>• Initials of the authors who received each award<br>• Grant numbers awarded to each author<br>• The full name of each funder<br>• URL of each funder website<br>• Did the sponsors or funders play any role in the study design, data collection and analysis, decision to publish, or preparation of the manuscript?<br><br>Did you receive funding for this work?	The author(s) received no specific funding for this work.
**Competing Interests**<br><br>Use the instructions below to enter a competing interest statement for this submission. On behalf of all authors, disclose any competing interests that	he authors have declared that no competing interests exist

could be perceived to bias this work—acknowledging all financial support and any other relevant financial or non-financial competing interests.<br><br>This statement is required for submission and **will appear in the published article** if the submission is accepted. Please make sure it is accurate and that any funding sources listed in your Funding Information later in the submission form are also declared in your Financial Disclosure statement.<br><br>View published research articles from *PLOS ONE* for specific examples.<br><br>**NO authors have competing interests**<br><br>Enter: *The authors have declared that no competing interests exist.*<br><br>**Authors with competing interests**<br><br>Enter competing interest details beginning with this statement:<br><br>*I have read the journal's policy and the authors of this manuscript have the following competing interests: [insert competing interests here]*<br><br>* typeset	
**Ethics Statement**<br><br>Enter an ethics statement for this submission. This statement is required if the study involved:<br><br>• Human participants<br>• Human specimens or tissue<br>• Vertebrate animals or cephalopods<br>• Vertebrate embryos or tissues<br>• Field research<br><br>Write "N/A" if the submission does not require an ethics statement.	Current work only involve clinical strains obtained by the typical procedures and guidelines, however, the strains used and the experimental plan was approved by the research committee of the Institute with the assigned number 55/22 AC.

Powered by Editorial Manager® and ProduXion Manager® from Aries Systems Corporation

General guidance is provided below. Consult the submission guidelines for detailed instructions. **Make sure that all information entered here is included in the Methods section of the manuscript.**<br><br>**Format for specific study types**<br><br>**Human Subject Research (involving human participants and/or tissue)**<br>• Give the name of the institutional review board or ethics committee that approved the study<br>• Include the approval number and/or a statement indicating approval of this research<br>• Indicate the form of consent obtained (written/oral) or the reason that consent was not obtained (e.g. the data were analyzed anonymously)<br><br>**Animal Research (involving vertebrate animals, embryos or tissues)**<br>• Provide the name of the Institutional Animal Care and Use Committee (IACUC) or other relevant ethics board that reviewed the study protocol, and indicate whether they approved this research or granted a formal waiver of ethical approval<br>• Include an approval number if one was obtained<br>• If the study involved *non-human primates*, add *additional details* about animal welfare and steps taken to ameliorate suffering<br>• If anesthesia, euthanasia, or any kind of animal sacrifice is part of the study, include briefly which substances and/or methods were applied<br><br>**Field Research**<br><br>Include the following details if this study involves the collection of plant, animal, or other materials from a natural setting:<br>• Field permit number<br>• Name of the institution or relevant body that granted permission	
**Data Availability**<br><br>Authors are required to make all data	Yes - all data are fully available without restriction

Powered by Editorial Manager® and ProduXion Manager® from Aries Systems Corporation

underlying the findings described fully available, without restriction, and from the time of publication. PLOS allows rare exceptions to address legal and ethical concerns. See the PLOS Data Policy and FAQ for detailed information.<br><br>A Data Availability Statement describing where the data can be found is required at submission. Your answers to this question constitute the Data Availability Statement and **will be published in the article**, if accepted.<br><br>**Important:** Stating 'data available on request from the author' is not sufficient. If your data are only available upon request, select 'No' for the first question and explain your exceptional situation in the text box.<br><br>Do the authors confirm that all data underlying the findings described in their manuscript are fully available without restriction?	
**Describe where the data may be found in full sentences. If you are copying our sample text, replace any instances of XXX with the appropriate details.**<br><br>• If the data are **held or will be held in a public repository**, include URLs, accession numbers or DOIs. If this information will only be available after acceptance, indicate this by ticking the box below. For example: *All XXX files are available from the XXX database (accession number(s) XXX, XXX.)*.<br>• If the data are all contained **within the manuscript and/or Supporting Information files**, enter the following: *All relevant data are within the manuscript and its Supporting Information files.*<br>• If neither of these applies but you are able to provide **details of access elsewhere**, with or without limitations, please do so. For example:<br><br>*Data cannot be shared publicly because of [XXX]. Data are available from the*	This Whole Genome Shotgun project has been deposited at DDBJ/ENA/GenBank (https://www.ncbi.nlm.nih.gov/bioproject/?term=PRJNA1094452) under the accession numbers JBBPHI000000000, JBBPHJ000000000, JBBPHK000000000, JBBPHL000000000, JBBPHM000000000, JBBPHN000000000 and JBBYHJ000000000. The version described in this paper are JBBPHI010000000, JBBPHJ010000000, JBBPHK010000000, JBBPHL010000000, JBBPHM010000000, JBBPHN010000000 and JBBYHJ010000000.

XXX Institutional Data Access / Ethics Committee (contact via XXX) for researchers who meet the criteria for access to confidential data.<br><br>*The data underlying the results presented in the study are available from (include the name of the third party and contact information or URL).*<br>• This text is appropriate if the data are owned by a third party and authors do not have permission to share the data.<br><br>* typeset	
Additional data availability information:	Tick here if the URLs/accession numbers/DOIs will be available only after acceptance of the manuscript for publication so that we can ensure their inclusion before publication.

Powered by Editorial Manager® and ProduXion Manager® from Aries Systems Corporation

Manuscript Click here to access/download;Manuscript;Myroides spp.docx

Myroides species, pathogenic spectrum and clinical microbiology sight in Mexican isolates.

Author list:

Claudia Adriana Colín-Castro[1¶], Josue Mizael Ortiz-Álvarez[2¶], Cindy Fabiola Hernández-Pérez[3], Melissa Hernández-Durán[1], María de Lourdes García-Hernández[1], María Guadalupe Martínez-Zavaleta[1], Noé Becerra-Lobato[1], Mercedes Isabel Cervantes-Hernández[1], Graciela Rosas-Alquicira[1], Guillermo Cerón-González[1], Braulio Josué Méndez-Sotelo[4], Rodolfo García-Contreras[5], Rafael Franco-Cendejas[6*], Luis Esaú López-Jácome[1,7*].

Affiliations:

[1] Clinical Microbiology Laboratory, Infectious Diseases Division. Instituto Nacional de Rehabilitación Luis Guillermo Ibarra Ibarra, Mexico City. Mexico.

[2] Programa “Investigadoras e Investigadores por México”. Consejo Nacional de Humanidades, Ciencias y Tecnologías (CONAHCYT), Mexico City, Mexico.

[3] Centro Nacional de Referencia de Inocuidad y Bioseguridad Agroalimentaria. Servicio Nacional de Sanidad, Inocuidad y Calidad Agroalimentaria (SENASICA). Tecámac, Mexico State.

[4] Infectious Diseases Division. Instituto Nacional de Rehabilitación Luis Guillermo Ibarra Ibarra, Mexico City. Mexico.

[5] Bacteriology Laboratory, Microbiology and Parasitology Department, Medicine Faculty, Universidad Nacional Autónoma de México, Mexico City, Mexico.

[6] Biomedical Research Subdirection, Instituto Nacional de Rehabilitación Luis Guillermo Ibarra Ibarra, Mexico City, Mexico.

[7] Biology Department, Chemistry Faculty, Universidad Nacional Autónoma de México, Mexico City, Mexico

Correspondence.

Luis Esaú López Jácome. Clinical Microbiology Laboratory, Instituto Nacional de Rehabilitación Luis Guillermo Ibarra Ibarra. Avenida México-Xochimilco 289 Col. Arenal de Guadalupe, Alcaldía Tlalpan, 14289, Ciudad de México (CDMX), México and Biology Department, Chemistry Faculty, Universidad Nacional Autónoma de México. Circuito Exterior S/N, Ciudad Universitaria, Alcaldía Coyoacán, 04510, Ciudad de México (CdMx), México. esaulopezjacome@quimica.unam.mx

Rafael Franco-Cendejas, Biomedical Research Subdirection, Instituto Nacional de Rehabilitación Luis Guillermo Ibarra Ibarra, Ciudad de México, Mexico. Av. México-Xochimilco #289 Col. Arenal de Guadalupe, Alc. Tlalpan. CP 14289, Mexico City, Mexico. raffcend@yahoo.com

Running title: Infections related to *Myroides* spp.

¶ Both authors participated equally

Keywords: *Myroides* spp., antibiotic resistance, infection, skin and soft tissue, pathogen.

ABSTRACT

Myroides is a bacterial genus of opportunistic bacteria responsible for diverse infections including in the skin and soft tissues, urinary tract, cardiovascular system, and bacteremia, although the incidence of his reported infections is low, it is increasing, likely due the use of better bacterial identification methods, but also perhaps due an increase in its prevalence.

Objective: To describe the microbiological and genetic characteristics of seven different *Myroides spp.* clinical strains and comment on their phylum, pathogenic and resistance characteristics.

Methods: *Myroides* spp., strains were identified by miniaturized biochemistry and MALDI-ToF. Susceptibility tests were performed according to CLSI recommendations by broth microdilution. Whole genome sequencing was performed for each strain and bioinformatics analysis were performed.

Results: Strains were identified at genus level by two methodologies. Our results revealed that likely four strains belong to the species *Myroides odoratimimus*, while the other two may be undescribed ones. Remarkably, all isolates harbored several genes encoding antibiotic resistance determinants for ß-lactams, aminoglycosides and glycopeptides and in concordance, presented high levels of resistance, against these antibiotics; moreover, the presences of carbapenemases were evidenced by meropenem degrading activity in five isolates and at least two strains possessed plasmids harboring mainly ribosomal RNA genes, tRNAs and genes encoding proteins with unknown functions.

Conclusions: Our study increases the knowledge about the biology of this understudied genus and highlights the potential of *Myroides* to emerge as a broader cause of recalcitrant opportunistic infections.

INTRODUCTION

The genus *Myroides,* first isolated in 1923 in feces of patients with gastrointestinal infections [1], belongs to phylum *Bacteroidota*, class *Flavobacteriia*, order *Flavobacteriales* and family *Flavobacteriaceae*. *Myroides* is comprised of 13 species in NCBI Taxonomy Browser, which comprise *Myroides albus, Myroides fluvii, Myroides gitamensis, Myroides guanonis, Myroides indicus, Myroides injenensis, Myroides marinus, Myroides odoratimimus, Myroides odoratus, Myroides oncorhynchi, Myroides pelagicus, Myroides phaeus* and *Myroides profundi* [2,3]. *Myroides* was initially included within *Flavobacterium* genus, specifically *Flavobacterium odoratum* and described by Stutzer in 1929, however, Vancanneyt and coworkers reclassified it and created a new genus, *Myroides* [4]. The given name came from Greek noun *mýron* which means perfume and Latin suffix *oides* means similar, shape, form or resembling (used in taxonomy), therefore *Myroides* resembling perfume, this due to the sweet odor, like fruits, characteristics in this genus [5]. It is an aerobic strict, thus is positive for oxidase and catalase activity, Gram-negative rod, 0.5 x 1-2 µm, non-motile, including gliding. Growing conditions characterizes its ability to grow on MacConkey agar and non-hemolysis production, growth at 18-22° C, and 37° C. *Myroides* produces, in the most of times, yellow or orange colonies [5].

Clinically, only four species of *Myroides* (*M. odoratimimus*, followed by *M. odoratus. M. injenensis* and *M. phaeus*) have been related with different infections such as endocarditis, pericarditis, urinary tract infections, skin and soft tissue infections, ventriculitis, liver abscesses and bacteremia, either in immunocompromised or immunocompetent host, as well [6–9]. The number of reported infections by *Myroides* species have been documented in a major frequency due the employment of more sensible and reliable techniques, such as matrix-assisted laser desorption/ionization time-of-flight mass spectrometry (MALDI-TOF

MS) and 16S ARN sequencing; *Myroides* species have been reported specially in hospital outbreaks causing catheter related urinary tract infections [10,11] associate, probable, to some factors such as prolonged use of antibiotics, immunosuppression, and invasive procedures [12]. Furthermore, it is linked to multiple antimicrobial resistance mechanisms [13]. However, as data expands, there must be more species associated with different infections that might be added, in this sense, information, either microbiological or genetic, is limited and impact of *Myroides spp.,* infections are not well understood, therefore, the role of infections is poorly documented. The aim of this work was to describe the microbiological and genetic characteristics of seven different *Myroides spp.* clinical strains and comment on their phylum, pathogenic and resistance characteristics.

MATERIAL AND METHODS

Clinical strains

We conducted a retrospective study seeking *Myroides spp.,* isolates from a tertiary care Hospital in Mexico City. We included all strains isolated from invasive samples, including blood cultures, lower respiratory samples, abscesses, and tissue biopsies, over an eleven-year period from 1/January/2012 to 1/January/2023 and were accessed for research on 15/May/2023 . Duplicated samples were excluded. The collection and sample processing were conducted at Clinical Microbiology Laboratory at Instituto Nacional de Rehabilitación Luis Guillermo Ibarra Ibarra in Mexico City. All isolates were stored at -70°C and subsequently inoculated onto 5% sheep blood agar prior to their use. Identification at the beginning was performed with the Vitek 2 compact (BioMérieux, Marcy ′Étoile, France) and was confirmed with Vitek MS (MALDI-ToF, (BioMérieux, Marcy ′Étoile, France)) following manufacturer's recommendations. Information from clinical records was obtained. Current work only involves clinical strains obtained by the typical procedures and guidelines,

however, the strains used and the experimental plan was approved by the research committee of the Institute with the assigned number 55/22 AC.

Minimal inhibitory concentrations determination

The antibiotics evaluated were: Amikacin (AK), Gentamicin (GN), Aztreonam (ATM), Ceftazidime (CAZ), Cefepime (FEP), Ciprofloxacin (CIP), Levofloxacin (LVX), Meropenem (MEM), Imipenem (IMP), Colistin (CL), Piperacillin/Tazobactam (TZP), azithromycin (AZT), Erythromycin (E), Doxycycline (DO), Tigecycline (TYG) and Sulfamethoxazole/trimethoprim (SXT) (Sigma Aldrich, Burlington, Massachusetts, USA). Broth microdilution assay was performed following the recommendations by the CLSI [19]. *E. coli* ATCC 25922 was used for quality control. Due breakpoints are not defined for *Myroides spp.,* neither in CLSI or EUCAST MIC was defined as the lowest concentration that inhibits bacterial growth.

Phenotypic test to determine carbapenemase activity.

Carbapenemase test was performed for each sample according to CLSI M100 2023 recommendation [19]. Briefly, colonies were harvested with a 10 µL loop and deposited into a 2 mL polypropylene tube with 1.5 mL Trypticase soy broth, then a 10 µg MEM disc added into the tube. The tubes were incubated during 4 h at 37° C. After that, *E. coli* ATCC 25922 pan susceptible at 0.5 McFarland Scale was inoculated onto Mueller-Hinton plates. The same strain of *E. coli* ATCC 25922 was used as negative control and *Klebsiella pneumoniae* ATCC BAA-1705 as positive control (carbapenemase producer). The interpretation of the test was according to CLSI M100 2023 [19].

Whole genome sequencing

Genomic DNA was extracted with DNeasy Blood & Tissue (QIAGEN). Once extracted, DNA quality and concentration were determined with the Qubit 3.0 fluorometer (Invitrogen, USA) and the Nanodrop One/One spectrophotometer (Thermo Fisher Scientific, USA) and samples were kept at -20°C, until use. Library preparation was performed using the Illumina DNA Prep (Illumina, USA) for the tagmentation, indexing and cleaning steps, as well as the employment of IDT for Illumina DNA/RNA UD Indexes (Illumina, USA) for the indexing step. Library quality control was performed using the Qubit 3.0 fluorometer (Invitrogen, USA) and the 4200 Tapestation System (Agilent, USA). Sequencing of pooled and normalized libraries was made using the MiSeq Reagent Kit V2 (300 cycles) on the Illumina MiSeq platform in a paired end configuration. Samples were sequenced at Centro Nacional de Referencia de Inocuidad y Bioseguridad Agroalimentaria from Servicio Nacional de Sanidad, Inocuidad y Calidad Agroalimentaria.

Plasmid extraction and purification.

In addition, plasmids were searched in all strains included. Plasmids were isolated and purified with the E.Z.N.A Plasmid DNA Mini Kit I (Omega bio-tek, Norcross, Georgia, USA). Once extracted, 5 µL of DNA were run in 1% agarose gel and visualized through Gel Doc XR+ with Image Lab Software (Bio-Rad; Hercules, California, USA), a 100 bp molecular ladder was used (Invitrogen; Waltham, Massachusetts, USA). Plasmid library preparation and sequencing were performed in the same manner as for genomic DNA.

Genomic analysis

The Quality of the Sequenced Read Archives (SRA) generated was analyzed by using FastQC [20]. The removal of contaminations and sequences with poor Phred values <30

was conducted with Trimmomatic [21]. The SRA of each strain were assembled with SPAdes [22]. Each assembling quality were determined with QUAST [23]. The contigs of each genome were subject to the removal of sequences with coverage values <200 by using the seqtk_seq included in the sektq package [24]. A general annotation for the contigs was performed with Prokka [25]. The evaluation of the annotation based on completeness determination was conducted by using BUSCO [26].

A preliminary identification of the strains was performed with the generation of a phylogenetic tree based on the analysis of partial 16S rRNA. The sequences belonging to the 16S rRNA gene of each sequenced genome for this study were extracted by using barrnap [27]. The sequences of type strains were obtained from the non-redundant Genbank database [28]. A multiple sequence alignment (MSA) of 16S sequences was obtained with MUSCLE [29]. The Maximum Likelihood (ML) phylogeny was constructed in IQTREE employing the TVM + F + I + G4 evolutionary model with Ultrafast-Bootstrap determination of 10,000 replicates [30]. The 16S-based phylogenetic trees were edited and visualized with iTOL v5 [31]. As complementary, pairwise gene identity scores were determined with Protologger [32].

The annotation of Antimicrobial Resistance Elements (ARE) was conducted with Abricate [33], by using the Comprehensive Antibiotic Resistance Database (CARD) [34]. The annotation of the virulence factors (VF) was performed by using Markov Hidden Models with HMMER package [35] and custom scripts, based on the VF identified for *M. odoratimimus* [36]. The plasmids were extracted and typed with MOB Suite [37] ; annotated and visualized with Proksee web server [38].

Data availability Statement

This Whole Genome Shotgun project has been deposited at DDBJ/ENA/GenBank (https://www.ncbi.nlm.nih.gov/bioproject/?term=PRJNA1094452) under the accession numbers JBBPHI000000000, JBBPHJ000000000, JBBPHK000000000, JBBPHL000000000, JBBPHM000000000, JBBPHN000000000 and JBBYHJ000000000. The version described in this paper are JBBPHI010000000, JBBPHJ010000000, JBBPHK010000000, JBBPHL010000000, JBBPHM010000000, JBBPHN010000000 and JBBYHJ010000000.

RESULTS

Strains included.

During the study period, a total of seven strains previously identified such as *Myroides spp.*, which were confirmed with Vitek 2 compact and with Vitek MS, such as *Myroides* spp. Four isolates affected males, five cases were associated with skin and deep tissue infections (Table 1).

Table 1. Clinical characteristics, treatment given, and outcomes of clinical strains included.

Number	Gender/ Age (years)	Comorbidities	Type of infection	Clinical sample	Isolation Date	Treatment	Outcome
EB1487	Male / 17	Congenital scoliosis	Catheter related Urinary tract infection	Urine	23/04/2014	Ciprofloxacin	Cured
C1519	Female / 37	Spinal cord injury and trauma	Osteomyelitis	Bone biopsy	06/08/2015	Meropenem	Cured
C1996	Female / 41	Burn injury	SSTI	Quantitative biopsy (skin)	14/11/2016	Meropenem	Died / Other infections
C2723	Male / 11	Trauma	SSTI	Quantitative biopsy (skin)	29/11/2018	Amikacin	Cured
C4067	Male / 8	Trauma	SSTI	Quantitative biopsy (skin)	15/07/2021	Ciprofloxacin	Cured
C4256	Male / 24	Burn injury	Osteomyelitis	Bone biopsy	28/09/2021	Meropenem	Cured
C4411	Female / 48	Ependymoma	Catheter related urinary tract infection	Urine	09/12/2021	None (catheter changed)	Cured

Treatment chosen was different for each strain including fluoroquinolones, aminoglycosides and carbapenems, meanwhile for osteomyelitis the antimicrobial selected was meropenem, with different time periods depending on the type of infection.

.

Susceptibility patterns

The greatest resistance rates were observed for aminoglycosides (AK and GN) with MICs ≥ 256 µg/mL, followed by ß-lactams (ATM, cephalosporins and TZP). On the other hand, azithromycin had lower MICs (around 2 µg/mL the most) in comparison with erythromycin which had the higher concentration with 4 µg/mL and the lowest with 0.5 µ/mL; however, tetracyclines showed similar MICs between members assessed. The lowest MICs observed in antibiotics evaluated were in SXT (Table 2A and 2B).

Table 2A. *Myroides spp.* Minimum inhibitory concentrations of clinical strains.

Strain	AK (µg/mL)	GN (µg/mL)	ATM (µg/mL)	CAZ (µg/mL)	FEP (µg/mL)	CIP (µg/mL)	LVX (µg/mL)	MEM (µg/mL)	IMP (µg/mL)	COL (µg/mL)	TZP (µg/mL)
EB1487	256	> 256	128	256	64	4	4	4	4	> 128	128/4
C1519	> 256	> 256	64	128	32	0.5	0.5	8	4	> 128	128/4
C1996	> 256	> 256	256	256	64	64	32	2	2	> 128	128/4
C2723	256	64	256	64	8	2	4	1	0.25	> 128	32/4
C4067	> 256	> 256	256	256	62	2	2	8	4	> 128	128/4
C4256	> 256	> 256	256	256	62	2	2	8	4	> 128	128/4
C4411	> 256	> 256	256	256	62	2	2	8	4	> 128	128/4

AK: amikacin; GN: gentamicin; ATM: aztreonam; CAZ: ceftazidime; FEP: cefepime; CIP: ciprofloxacin; LVX: levofloxacin; MEM: meropenem; IMP: imipenem; COL: colistin; TZP: piperacillin/tazobactam

Table 2B. *Myroides spp.* Minimum inhibitory concentrations.

Strain	AZT (µg/mL)	E (µg/mL)	DOX (µg/mL)	TIG (µg/mL)	SXT (µg/mL)
EB1487	1	4	0.5	2	1/19
C1519	1	0.5	0.125	1	0.5/9.5
C1996	2	4	2	2	1/19
C2723	0.5	2	0.5	0.25	2/36
C4067	2	2	1	0.5	1/19
C4256	2	4	1	0.5	1/19
C4411	2	2	1	0.5	2/36

AZT: azithromycin; DOX: doxycycline; E: erythromycin; TIG: tigecycline; SXT: sulfamethoxazole/trimethoprim.

Phenotypic test to determine carbapenemase activity.

Due to high MICs values for ß-lactam antibiotics, including carbapenem, we performed phenotypic assay looking for carbapenemases presence according to CLSI guidelines M100, however, this procedure is not yet validated and standardized for microorganisms different to *Enterobacterales* or *Pseudomonas aeruginosa*. We identified hydrolytic activity against MEM in EB1487, C1519, C4411 with halos < 15 mm, on the other hand, C1996 and C4256 had halos with 16 mm, according CLSI M100 definitions, for *Enterobacterales* and *Pseudomonas* this measure correspond with the definition of inconclusive, while the rest of

the strains had halos > 19 mm (negative); however, as mentioned above, this is not a validated strategy for this microorganism, so the negative result could be due to a limitation of the strategy.

Molecular identification

The phylogenetic tree reconstructed exhibited the following: four strains (C4366, EB1487, C4411 and C1996) were clustered with *M. odoratimimus* type strain, with an identity percentage > 99% (Figure 1) (Table 3). Hence, the results indicate that these four strains may be identified as *M. odoratimimus*. By the other hand, the strain C4067 evidenced a phylogenetic relationship with *Myroides marinus*, however, they shared a 95.67 % of identity. Likewise, the strains C2723 and C1519 were clustered in the same clade with *Myroides phaeus*, showing a 96.3 % of identity among them; consequently, we suspect the strains of *Myroides* C4067, C2723 and C1519 may be cataloged as new species.

Table 3. Sequences of study for ML phylogenetic tree reconstruction.

Organism	GenBank ID
Myroides odoratus	M58777
Myroides fluvii	MK129421
Myroides pelagicus	AB176662
Myroides albus	MK734183
Myroides injenensis	HQ671078
Myroides phaeus	GU253339
Myroides oncorhynchi	OL437262
Myroides profundi	EU204978
Myroides odoratimimus	AJ854059
Myroides gitamensis	HF571338
Myroides marinus	GQ857652
Flavobacterium olei	KX672808
Flavobacterium azizsancarii	OQ024221
Flavobacterium soyangense	KX061439

Flavobacterium dasani	MH019224
Flavobacterium aquaticum	HE995762
Flavobacterium vireti	KM576853
Flavobacterium inkyongense	KX025140
Flavobacterium fulvum	KU052686
Flavobacterium yanchengense	JX548325
Flavobacterium ardleyense	KX911209
Flavobacterium aquimarinum	KY612936
Flavobacterium ponti	GQ370387
Flavobacterium shanxiense	KJ641612
Cytophaga massiliensis	EF394924
Flavobacterium naphthae	MF405102
Myroides cloacae	KU746272
Myroides anatoliensis	JF825522

Virulence Factors (VF) and Antimicrobial Resistance (AMR) genes detection

A total of 15 elements associated with VF and classified in seven categories were recognized and annotated. The summary of this result is displayed in Figure 2A. All elements were identified in all strains, nevertheless *sodB* (encoding for Superoxide dismutase) was not identified in the isolates C1519 and C2723, whereas *wecC*, encoding for UDP-N-acetyl-D-mannosaminuronic acid dehydrogenase was not detected in the isolate EB1487. The VF recognized maybe associated with production of capsular polysaccharide, cell wall peptidoglycan, heme biosynthesis (oxidative respiration), intracellular survival factors (ISF), chaperones, streptococcal enolase and type 4 secretion system effectors (T4SS effectors).

Also 8 kinds of elements associated with Antibiotic Resistance (ARE) were detected (Figure 2B). The strains showed an average of about 15 AMR genes, exhibiting an evident multidrug resistance and harboring high diversity of AREs. The isolate *Myroides spp*. C4067 presented the highest diversity and content of AREs, with a total of 22 genes. On the other hand, the strain *Myroides spp*. C2723 exhibited the lowest content of AMR genes. In general, the drug class assigned for the AMR genes identified was congruent with the MIC profiles obtained in experimental assays. Even though the *tet(X2)* gene was identified in two strains, these displayed susceptibility to tetracyclines. Also, the *iri* and *rphA*, linked with rifampicin resistance were detected. The strains exhibited a significant abundance of AREs associated with ß-lactam, aminoglycosides, and glycopeptides resistance, matching with the resistance profiles to this antibiotic observed. Interestingly, all strains showed the presence of *ereB*, an erythromycin esterase-like associated with resistance to macrolides, despite the strains being susceptible to azithromycin. Interestingly, strains with GOB-16, a metallo ß-lactamase, were C4256, EB1487, C4067, C4411, these same strains shared another metallo ß-lactamase belonging to the IMP family (IMP-27), strain C1996 carried IMP-27 but not GOB-16. On the other hand, strains C4411 and C4256 co-carried two IMPs (IMP-27 and IMP-35) in addition to GOB-16. MUS-1 is a metallo ß-lactamase identified in *Myroides* present in almost all isolates except C2723 and C1519. Of the members of the oxacillinase (OXA) family with carbapenemase activity we found OXA-229 (C4411, C4067 and C4256), OXA-351 (C4067) and OXA-97 (C4256). Of the AmpC group, PDC-85 was found in C2723 and C1519. Genes associated with colistin resistance were found in *mcr3.10, mcr3.6* and *mcr3.7*. Strains C1996, C4256, C4067 shared the presence of *mcr3.6* and *mcr3.7* genes. EB1487 co-carried *mcr3.10* and *mcr3.6* while C4411 carried only *mcr3.7*. Phenotypically, EB1487, C1519 and C4411 showed carbapenemase activity against meropenem.

Plasmid identification and annotation

Two plasmids were detected (Figure S1) in the strains *Myroides* spp. C2723 (Plasmid ID C2723_C6) and *M. orodatimimus* EB1487 (Plasmid ID EB1487_S7) (Figure 3). The C2723_C6 showed an approximate size of 19 Kb, whereas EB1487_S7 exhibited a size of 150 Kb. Therefore, we consider EB1487_S7 as a megaplasmid. The annotation of the plasmid showed that C2723_C6 harbors ribosomal RNA genes, as well as tRNA sequences. A total of 11 proteins were predicted, however, the annotation assigned them as hypothetical proteins. By the other hand, the EB1487_S7 also harbored ribosomal RNA and tRNA genes, and 101 proteins were predicted, of which 75 were cataloged as hypothetical proteins. Twenty-six genes were properly annotated for EB1487_S7 whose functional classification may be associated with mobile elements, DNA processing, amino acid metabolism, aerobic respiration, cell wall peptidoglycan biosynthesis, carbon metabolism, ROS defense, and membrane transport.

DISCUSSION

Since the reclassification of *Flavobacterium odoratum* and the creation of the new genus *Myroides* by Dr. Vancanneyt M et al. in 1996 [4], very little evidence has been generated on the role of *Myroides* associated with infections in patients, only 43 articles were found in Pubmed since 2000 using the search terms "*Myroides*" and "infection". Of these, bacteremia and urinary tract infection were the most frequent. The most common species were *M. odoratimimus* and *M. odoratus* [6,7,40–49,8,50–59,10,60–69,11,70,71,14–17,39]

Myroides species are typically found in immunocompromised hosts, although there have been a few cases reported in immune competent individuals [6,39,40]. In our series, skin, and soft tissue infections, as well as deep infections, were the most common. This is likely

since we are a tertiary care hospital specializing in orthopedics, rehabilitation, and burns. Except for one patient, all were successfully treated despite having various comorbidities. The deceased patient had burn injuries and was infected with *Myroides*, along with other Gram-negative bacteria. Burn patients are severely immunosuppressed, making them more susceptible to infections due to the loss of their skin barrier. No previous cases of *Myroides* infection have been reported in burned patients.

The identification of rutinary cultures within a clinical microbiology lab is performed with miniaturized biochemistry systems such as Vitek 2 [72] (BioMérieux, Marcy ′Étoile, France) and Phoenix (Becton-Dickinson, New Jersey, USA) [50]. The introduction of instruments with larger databases and better precision and sensitivity, such as MALDI-ToF, has increased detection capacity, but even these types of systems, have methodological limitations for a suitable discrimination among species [73,74]. Therefore, we recurred to a presumptive molecular identification of pathogens based on phylogenetic analysis of 16S rRNA. The phylogenetic reconstruction has been useful for other studies for resolving properly the identity of isolates belonging to *Stenotrophomonas* genus [75]. The use of 16S analysis as a robust tool for identification in the clinical routine is not common [76], but has been widely explored in clinical research studies for a presumptive assignment of identity to bacteria species [77–80]. According to the criteria for assignment of bacterial species established by Roselló Mora [81], based on 16S-rRNA identity percentage (>97%). We proposed that four isolated species were designated as *M. odoratimimus*, while the strains C2723, C1519 and C4067 may be cataloged as new species of *Myroides*. The phylogenetic evidence as a robust tool for identification supports our predictions. Nonetheless, further studies based on a more rigorous genome-based phylogenetic analysis are needed to validate our hypothesis.

Due to the broad AMR spectrum observed in the isolates of study, we decided to identify the ARE presumptively involved in pan-drug resistance. For instance, previous reports describe that *M. odoratimimus* may harbor up to 32 AMR genes [36,82,83] of which ß-lactamases and efflux pumps are the most abundant AREs observed. From 2014 until now, the main AREs identified for *Myroides* spp. has been the following: ß-lactamases: bla_{VIM}, bla_{IMP}, bla_{NMD}, bla_{OXA-78}, $bla_{OXA-209}$, $bla_{OXA-347}$, bla_{KPC}, bla_{TUS}, bla_{EBR-1}, bla_{MOC} and bla_{MUS-1}; tetracycline resistance: *tet(X6);* efflux pumps*: abeS, msrB, qacH, rosA;* macrolides: *erm(F), ere(D)* [12,36,61,84–86]. Here, we were able to detect the AREs aforementioned; however, it is important to highlight that the isolates analyzed in this work also displayed AREs and a phenotype associated with resistance to colistin, glycopeptides and rifampicin, a resistance trait not observed previously. These results suggest that the resistome of *Myroides* spp. is expanding remarkably, and probably soon the species of this genus might convert into an important health threat.

Other important trait is that multidrug resistance genotype was observed in the presumptive new species, although only *M. orodatimimus*, *M. odoratus, M. phaeus* and *M. injerensis* have been cataloged as relevant AMR pathogens [52,63,83,84], our results indicate that the multidrug resistance phenotype[illegible]ssibly is highly expanded and extended in several species of *Myroides*. In the case of *M. orodatimimus* a chromosomic metallo ß-lactamase has been ~~describe~~, the MUS-1 [87]. Two strains had an undetermined phenotype, however carrier, this due the phenotypic assay is not validated for bacteria different to *Enterobacterales* or *Pseudomonas* spp,

Pathogen species of *Myroides* present several VF associated with cell adherence, intracellular surveillance and capsule production [36,83]. Nonetheless, a more robust study of the molecular mechanism of pathogenicity of *Myroides* spp. have not been performed yet. Thus, currently we do not have enough information about how *Myroides* infects and causes

disease to the host. According to the VF identified, these results suggest that the isolates of studies exhibit capability of intracellular surveillance and protection against phagocytic cells of the immune system, a virulence trait observed in other opportunistic pathogens such as *Mycobacterium spp*, *Pseudomonas spp*, *Serratia spp,* etc [88–90].

CONCLUSIONS

Myroides genus is not such as uncommon microorganism causing infections, both in immunosuppressed and immune competent hosts, with different outcomes depending on antimicrobial profiles. Molecular techniques are important to identify properly these bacteria, characterize more possible species and explore therapeutic options for achieving clearance of its infections.

STATEMENTS AND DECLARATIONS

CONFLICT OF INTEREST

We, the authors, declare for the present work, in all honesty, that we have no conflict of interest.

FUNDING

In current work we did not receive any economical support or any external funding.

ACKNOWLEDGMENTS

To SENASICA and WHO Collaborating Centre on Antimicrobial Resistance in Foodborne and Environmental Bacteria (MEX-33), especially Mayrén Zamora Nava and all the sequencing and bioinformatics staff for helping us to sequence our samples.

AUTHOR CONTRIBUTION

Claudia Adriana Colín-Castro[1¶], Jossue Ortiz-Álvarez[2¶], Cindy Fabiola Hernández-Pérez[3], Melissa Hernández-Durán[1], María de Lourdes García-Hernández[1], María Guadalupe Martínez-Zavaleta[1], Noé Becerra-Lobato[1], Mercedes Isabel Cervantes-Hernández[1], Graciela Rosas-Alquicira[1], Guillermo Cerón-González[1], Braulio Josué Méndez-Sotelo[4], Rodolfo García-Contreras[5], Rafael Franco-Cendejas[6*], Luis Esaú López-Jácome[1,7*].

Conceptualization: Franco-Cendejas Rafael and López-Jácome Luis Esaú

Formal Analysis: Claudia Adriana Colín-Castro, Cindy Fabiola Hernández-Pérez, Méndez-Sotelo Braulio J., Hernández-Durán Melissa, María de Lourdes García-Hernández, María Guadalupe Martínez-Zavaleta, Noé Becerra-Lobato, Mercedes Isabel Cervantes-Hernández, Graciela Rosas-Alquicira, Guillermo Cerón-González Ortiz-Álvarez Jossue, García-Contreras Rodolfo, Franco-Cendejas Rafael and López-Jácome Luis Esaú.

Methodology and running experiments: Claudia Adriana Colín-Castro, Hernández-Durán Melissa, Cindy Fabiola Hernández-Pérez, Méndez-Sotelo Braulio J., María

de Lourdes García-Hernández, María Guadalupe Martínez-Zavaleta, Noé Becerra-Lobato, Mercedes Isabel Cervantes-Hernández, Graciela Rosas-Alquicira, Guillermo Cerón-González, Jossue Mizael Ortiz-Álvarez, García-Contreras Rodolfo.

Supervision: Franco-Cendejas Rafael and López-Jácome Luis Esaú

Writing – original draft: Claudia Adriana Colín-Castro, Jossue Mizael Ortiz-Álvarez, Rafael Franco-Cendejas and Luis Esaú López-Jácome

Writing – review and editing: Claudia Adriana Colín-Castro, Jossue Mizael Ortiz-Álvarez, García-Contreras Rodolfo, Franco-Cendejas Rafael and López-Jácome Luis Esaú.

References

[1] Stutzer MJ. Zur Frage Über die Fäulnisbakterien im Darm. Zentralbl Bakteriol Parasitenkd Infekt Hyg 1923;91:87–90.

[2] Schoch CL, Ciufo S, Domrachev M, Hotton CL, Kannan S, Khovanskaya R, et al. NCBI Taxonomy: A comprehensive update on curation, resources and tools. vol. 2020. Oxford University Press; 2020. https://doi.org/10.1093/database/baaa062.

[3] NCBI. Taxonomy browser (Myroides). Myroides 2023. https://www.ncbi.nlm.nih.gov/Taxonomy/Browser/wwwtax.cgi (accessed December 20, 2023).

[4] Vancanneyt M, Segers P, Torck U, Hoste B, Bernardet J-F, Vandamme P, et al. Reclassification of Flavobacterium odoratum (Stutzer 1929) Strains to a New Genus, Myroides, as Myroides odoratus comb. nov. and Myroides odoratimimus sp.

nov. Int J Syst Bacteriol 1996;46:926–32. https://doi.org/10.1099/00207713-46-4-926.

[5] Bergey’s Manual® of Systematic Bacteriology. Springer New York; 2010. https://doi.org/10.1007/978-0-387-68572-4.

[6] Benedetti P, Rassu M, Pavan G, Sefton A, Pellizzer G. Septic shock, pneumonia, and soft tissue infection due to Myroides odoratimimus: report of a case and review of Myroides infections. Infection 2011;39:161–5. https://doi.org/10.1007/s15010-010-0077-1.

[7] Lu Y, Xia W, Zhang X, Ni F, Mei Y. A Confirmed Catheter-Related Blood Stream Infection (CRBSI) in an Immunocompetent Patient Due to Myroides odoratimimus: Case Report and Literature Review. Infect Drug Resist 2020;Volume 13:139–44. https://doi.org/10.2147/IDR.S234778.

[8] Prateek S. Fatal Case of Pericardial Effusion Due to Myroides Odoratus : A Rare Case Report. J Clin DIAGNOSTIC Res 2015. https://doi.org/10.7860/JCDR/2015/15120.6740.

[9] Bachman KH, Sewell DL, Strausbaugh LJ. Recurrent Cellulitis and Bacteremia Caused by Flavobacterium odoratum. Clin Infect Dis 1996;22:1112–3. https://doi.org/10.1093/clinids/22.6.1112.

[10] Ktari S, Mnif B, Koubaa M, Mahjoubi F, Ben Jemaa M, Mhiri MN, et al. Nosocomial outbreak of Myroides odoratimimus urinary tract infection in a Tunisian hospital. J Hosp Infect 2012;80:77–81. https://doi.org/10.1016/j.jhin.2011.09.010.

[11] Yağcı A, ÇerikÇioğlu N, Kaufmann ME, Malnick H, Söyletir G, Babacan F, et al. Molecular Typing of Myroides odoratimimus (Flavobacterium odoratum) Urinary

Tract Infections in a Turkish Hospital. Eur J Clin Microbiol Infect Dis 2000;19:731–2. https://doi.org/10.1007/s100960070001.

[12] Aygar IS, Aydogan CN, Ozcan H, Unat I, Fatsa T, Tekin K, et al. Myroides odoratimimus: a New Threat with Persistent Infections, Multidrug Resistance, and the Potential for Hospital Outbreaks. Jpn J Infect Dis 2023;76:JJID.2023.005. https://doi.org/10.7883/yoken.JJID.2023.005.

[13] Gunzer F, Rudolph WW, Bunk B, Schober I, Peters S, Müller T, et al. Whole-genome sequencing of a large collection of Myroides odoratimimus and Myroides odoratus isolates and antimicrobial susceptibility studies. Emerg Microbes Infect 2018;7:1–8. https://doi.org/10.1038/s41426-018-0061-x.

[14] Khan U, Pandey E, Gandham N, Das N, Mukhida S, Kannuri S, et al. A case series and literature review of infections due to Myroides spp.: identification of contributing factors and emerging antibiotic susceptibility trends. Access Microbiol 2023;5. https://doi.org/10.1099/acmi.0.000549.v2.

[15] Bryan T. Green, Kevin Green P. Myroides odoratus Cellulitis and Bacteremia: Case Report and Review. Scand J Infect Dis 2001;33:932–4. https://doi.org/10.1080/00365540110077065.

[16] Paek J, Shin JH, Shin Y, Park I-S, Jin T-E, Kook J-K, et al. Myroides injenensis sp. nov., a new member isolated from human urine. Antonie Van Leeuwenhoek 2015;107:201–7. https://doi.org/10.1007/s10482-014-0317-y.

[17] Pérez-Lazo G, Morales-Moreno A, Soto-Febres F, Jove-Químper H, Morales-Castillo L, Palomares-Reyes C, et al. First report of Myroides phaeus bacteraemia identified by Polymerase chain reaction and genetic sequencing. IDCases 2020;19:e00695. https://doi.org/10.1016/j.idcr.2020.e00695.

[18] Yan S, Zhao N, Zhang X-H. Myroides phaeus sp. nov., isolated from human saliva, and emended descriptions of the genus Myroides and the species Myroides profundi Zhang et al. 2009 and Myroides marinus Cho et al. 2011. Int J Syst Evol Microbiol 2012;62:770–5. https://doi.org/10.1099/ijs.0.029215-0.

[19] Institute C and LS. CLSI. M100. Performance Standards for Antimicrobial Susceptibility Testing; Twenty-Second Informational Supplement. 33rd ed. Clinical and Laboratory Standars Institute; 2023.

[20] Simon Andrews. Babraham Bioinformatics - FastQC A Quality Control tool for High Throughput Sequence Data. Soil 2020;5:47–81.

[21] Bolger AM, Lohse M, Usadel B. Trimmomatic: a flexible trimmer for Illumina sequence data. Bioinformatics 2014;30:2114–20. https://doi.org/10.1093/bioinformatics/btu170.

[22] Bankevich A, Nurk S, Antipov D, Gurevich AA, Dvorkin M, Kulikov AS, et al. SPAdes: A New Genome Assembly Algorithm and Its Applications to Single-Cell Sequencing. J Comput Biol 2012;19:455–77. https://doi.org/10.1089/cmb.2012.0021.

[23] Gurevich A, Saveliev V, Vyahhi N, Tesler G. QUAST: quality assessment tool for genome assemblies. Bioinformatics 2013;29:1072–5. https://doi.org/10.1093/bioinformatics/btt086.

[24] Seatk: Toolkit for processing sequences in FASTA/Q formats. [https://github.com/lh3/seqtk] n.d.

[25] Seemann T. Prokka: rapid prokaryotic genome annotation. Bioinformatics 2014;30:2068–9. https://doi.org/10.1093/bioinformatics/btu153.

[26] Simão FA, Waterhouse RM, Ioannidis P, Kriventseva E V., Zdobnov EM. BUSCO: assessing genome assembly and annotation completeness with single-copy orthologs. Bioinformatics 2015;31:3210–2. https://doi.org/10.1093/bioinformatics/btv351.

[27] Torsten Seemann. tseemann/barrnap: :microscope: Bacterial ribosomal RNA predictor. GitHub 2018. https://github.com/tseemann/barrnap (accessed April 12, 2024).

[28] Sayers EW, Cavanaugh M, Clark K, Ostell J, Pruitt KD, Karsch-Mizrachi I. GenBank. Nucleic Acids Res 2019. https://doi.org/10.1093/nar/gkz956.

[29] Edgar RC. MUSCLE: multiple sequence alignment with high accuracy and high throughput. Nucleic Acids Res 2004;32:1792–7. https://doi.org/10.1093/nar/gkh340.

[30] Nguyen L-T, Schmidt HA, von Haeseler A, Minh BQ. IQ-TREE: A Fast and Effective Stochastic Algorithm for Estimating Maximum-Likelihood Phylogenies. Mol Biol Evol 2015;32:268–74. https://doi.org/10.1093/molbev/msu300.

[31] Letunic I, Bork P. Interactive Tree Of Life (iTOL) v5: an online tool for phylogenetic tree display and annotation. Nucleic Acids Res 2021;49:W293–6. https://doi.org/10.1093/nar/gkab301.

[32] Hitch TCA, Riedel T, Oren A, Overmann J, Lawley TD, Clavel T. Automated analysis of genomic sequences facilitates high-throughput and comprehensive description of bacteria. ISME Commun 2021;1. https://doi.org/10.1038/s43705-021-00017-z.

[33] Seeman T. GitHub - tseemann/abricate: :mag_right: Mass screening of contigs for antimicrobial and virulence genes. GitHub 2020.

https://github.com/tseemann/abricate (accessed April 12, 2024).

[34] Alcock BP, Raphenya AR, Lau TTY, Tsang KK, Bouchard M, Edalatmand A, et al. CARD 2020: antibiotic resistome surveillance with the comprehensive antibiotic resistance database. Nucleic Acids Res 2019. https://doi.org/10.1093/nar/gkz935.

[35] Eddy SR. Accelerated Profile HMM Searches. PLoS Comput Biol 2011;7:e1002195. https://doi.org/10.1371/journal.pcbi.1002195.

[36] Hu S, Cao L, Wu Y, Zhou Y, Jiang T, Wang L, et al. Comparative genomic analysis of Myroides odoratimimus isolates. Microbiologyopen 2019;8. https://doi.org/10.1002/mbo3.634.

[37] Robertson J, Nash JHE. MOB-suite: software tools for clustering, reconstruction and typing of plasmids from draft assemblies. Microb Genomics 2018;4. https://doi.org/10.1099/mgen.0.000206.

[38] Grant JR, Enns E, Marinier E, Mandal A, Herman EK, Chen C, et al. Proksee: in-depth characterization and visualization of bacterial genomes. Nucleic Acids Res 2023;51:W484–92. https://doi.org/10.1093/nar/gkad326.

[39] Bachmeyer C, Entressengle H, Khosrotehrani K, Goldman G, Delisle F, Arlet G, et al. Cellulitis due to Myroides odoratimimus in a patient with alcoholic cirrhosis. Clin Exp Dermatol 2007:071202194819001-??? https://doi.org/10.1111/j.1365-2230.2007.02590.x.

[40] Maraki S, Sarchianaki E, Barbagadakis S. Myroides odoratimimus soft tissue infection in an immunocompetent child following a pig bite: case report and literature review. Brazilian J Infect Dis 2012;16:390–2. https://doi.org/10.1016/j.bjid.2012.06.004.

[41] Deepa R, Venkatesh K, Parveen JD, Banu ST, Jayalakshmi G. Myroides odoratus and Chryseobacterium indologenes: Two rare isolates in the immunocompromised. Indian J Med Microbiol 2014;32:327–30. https://doi.org/10.4103/0255-0857.136592.

[42] Crum-Cianflone NF, Matson RW, Ballon-Landa G. Fatal case of necrotizing fasciitis due to Myroides odoratus. Infection 2014;42:931–5. https://doi.org/10.1007/s15010-014-0626-0.

[43] Endicott-Yazdani TR, Dhiman N, Benavides R, Spak CW. Myroides Odoratimimus Bacteremia in a Diabetic Patient. Baylor Univ Med Cent Proc 2015;28:342–3. https://doi.org/10.1080/08998280.2015.11929268.

[44] Lahmer T, Beitz A, Ehmer U, Schmid RM, Huber W. Septic shock due to Myroides odoratus in a medical intensive care unit patient with severe necrotising pancreatitis. Anaesth Intensive Care 2016;44:298–9.

[45] Jover-Sáenz A, Pérez-Villar F, Barcenilla-Gaite F. Sepsis grave por infección de prótesis articular por Myroides odoratimimus. Med Clin (Barc) 2016;147:276–7. https://doi.org/10.1016/j.medcli.2016.04.011.

[46] Ali MJ, Joseph J, Sharma S, Naik MN. Canaliculitis With Isolation of Myroides Species. Ophthalmic Plast Reconstr Surg 2017;33:S24–5. https://doi.org/10.1097/IOP.0000000000000604.

[47] Willems P, Muller J, Verhaegen J, Saegeman V, Desmet S. How to treat a fulminant erysipelas and sepsis caused by Myroides odoratimimus : case report and literature review. Acta Clin Belg 2017;72:331–5. https://doi.org/10.1080/17843286.2016.1245173.

[48] Pompilio A, Galardi G, Gherardi G, Verginelli F, Geminiani C, Pilloni AP, et al.

Infection of recurrent calcaneal ulcer caused by a biofilm-producer Myroides odoratimimus strain. Folia Microbiol (Praha) 2018;63:203–7. https://doi.org/10.1007/s12223-017-0552-5.

[49] Licker M, Sorescu T, Rus M, Cirlea N, Horhat F, Jurescu C, et al. Extensively drug-resistant Myroides odoratimimus – a case series of urinary tract infections in immunocompromised patients. Infect Drug Resist 2018;Volume 11:743–9. https://doi.org/10.2147/IDR.S161069.

[50] Lorenzin G, Piccinelli G, Carlassara L, Scolari F, Caccuri F, Caruso A, et al. Myroides odoratimimus urinary tract infection in an immunocompromised patient: an emerging multidrug-resistant micro-organism. Antimicrob Resist Infect Control 2018;7:96. https://doi.org/10.1186/s13756-018-0391-4.

[51] Ahamed I, Annapandian V, Muralidhara K. Myroides odoratimimus urinary tract infection. Saudi J Kidney Dis Transplant 2018;29:1220. https://doi.org/10.4103/1319-2442.243957.

[52] LaVergne S, Gaufin T, Richman D. Myroides injenensis Bacteremia and Severe Cellulitis. Open Forum Infect Dis 2019;6. https://doi.org/10.1093/ofid/ofz282.

[53] Meyer A, Dang H, Roland W. Myroides spp. cellulitis and bacteremia: A case report. IDCases 2019;18:e00638. https://doi.org/10.1016/j.idcr.2019.e00638.

[54] Choudhary M, Choudhary BK, Bera BC, Chaudhari SP, Giri DK, Ghosh RC, et al. Association of Myroides odoratimimus in immunocompromized piglets with post weaning multisystemic wasting syndrome. J Appl Microbiol 2019;127:1635–45. https://doi.org/10.1111/jam.14448.

[55] Yang S, Liu Q, Shen Z, Wang H, He L. Molecular Epidemiology of Myroides

odoratimimus in Nosocomial Catheter-Related Infection at a General Hospital in China. Infect Drug Resist 2020;Volume 13:1981–93. https://doi.org/10.2147/IDR.S251626.

[56] Kutlu HH, Avcı M, Dal T, Arı O, Durmaz R. A healthcare-associated outbreak of urinary tract infections due to Myroides odoratimimus. Jpn J Infect Dis 2020;73:421–6. https://doi.org/10.7883/yoken.JJID.2019.536.

[57] Foo RM, Nanavati SM, Samuel A, Lamm R, Upadhyay S. Gardener's Nightmare: A Rare Case of Myroides-Induced Septic Shock. Cureus 2020. https://doi.org/10.7759/cureus.12235.

[58] Beathard WA, Pickering A, Jacobs M. Myroides cellulitis and bacteremia: A case report. IDCases 2021;24:e01061. https://doi.org/10.1016/j.idcr.2021.e01061.

[59] Cruz-Choappa R, López E, Ocara M, Leiva Y. Myroides odoratimimus, una causa infrecuente de infección de tejidos blandos y osteomielitis. Rev Chil Infectología 2021;38:297–9. https://doi.org/10.4067/S0716-10182021000200297.

[60] Vempuluru V, Mitra S, Tripathy D, Mohapatra S, Rath S. Isolation of unusual bacteria in canaliculitis: A series of four cases. Saudi J Ophthalmol 2021;35:66. https://doi.org/10.4103/1319-4534.325778.

[61] Kurt AF, Mete B, Houssein FM, Tok Y, Kuskucu MA, Yucebag E, et al. A pan-resistant Myroides odoratimimus catheter-related bacteremia in a COVID-19 patient and review of the literature. Acta Microbiol Immunol Hung 2022. https://doi.org/10.1556/030.2022.01702.

[62] Faraz A, Fathima K, Kazmi SY, Al Motery AS, Ghaffar U Bin, Farhan MA. Recurrent Urinary Tract Infection in a Renal Transplant Patient by Pan-resistant Myroides Spp.

J Coll Physicians Surg Pak 2022;32:S34–6. https://doi.org/10.29271/jcpsp.2022.Supp1.S34.

[63] O'Neal M, Labay CE, Harris JE, Musick WL, Cernoch PL, Grimes KA, et al. Extensively Drug-Resistant Myroides odoratus in Critically Ill Patients: A Case Series and Literature Review. Case Rep Infect Dis 2022;2022:1–7. https://doi.org/10.1155/2022/6422861.

[64] Alberola Romano A, Gómez-Camarasa C, Pérez Rodríguez L, Vázquez Blanquiño A, Chueca Porcuna N. Myroides odoratus as an unusual urinary tract infection pathogen in immunosuppressed patient. Rev Española Quimioter 2022. https://doi.org/10.37201/req/045.2022.

[65] Jiang L-Z, Shen Y, Liang F, Ye X-M, Chen J, Yu Y-M. Intracranial Myroides odoratimimus Infection After EVD Successfully Treated with Intravenous Plus Intraventricular Tigecycline: A Case Report. Infect Drug Resist 2023;Volume 16:1955–63. https://doi.org/10.2147/IDR.S403088.

[66] Echevarría-Castro N, Matayoshi-Pérez A, Silva-Parra KA, Rojo-García D, Cucho Espinoza C. Cutaneous Infection Associated With Myroides odoratimimus Bacteremia in a Diabetic Patient. Cureus 2023. https://doi.org/10.7759/cureus.41328.

[67] Do S, Rebentish A, Ravichandran Kumar P. Case Report of Myroides odoratimimus Cellulitis in Chronic Venous Stasis Dermatitis With Literature Review. Cureus 2023. https://doi.org/10.7759/cureus.45319.

[68] Chapman C, Meeks B, Ung L, Nichols J. A Case of Myroides odoratimimus Bacteremia due to a Tunneled Dialysis Catheter-Associated Infection in a Paraplegic Patient. Case Rep Infect Dis 2023;2023:1–3.

https://doi.org/10.1155/2023/3089837.

[69] Sahu C, Patel SS, Chaudhary R, Bhartiya C, Bhatnagar N. A Retrospective Study on UTI by Myroides Species: An Emerging Drug Resistant Nosocomial Pathogen. Indian J Crit Care Med 2024;28:399–403. https://doi.org/10.5005/jp-journals-10071-24683.

[70] Belloir L, Billy PA, Hentgen C, Fille A, Barrans A. Bactériémie à Myroides odoratimimus. Médecine Mal Infect 2016;46:396–7. https://doi.org/10.1016/j.medmal.2016.05.003.

[71] Beharrysingh R. Myroides bacteremia : A case report and concise review. IDCases 2017;8:34–6. https://doi.org/10.1016/j.idcr.2017.02.012.

[72] Schröttner P, Rudolph WW, Eing BR, Bertram S, Gunzer F. Comparison of VITEK2, MALDI-TOF MS, and 16S rDNA sequencing for identification of Myroides odoratus and Myroides odoratimimus. Diagn Microbiol Infect Dis 2014;79:155–9. https://doi.org/10.1016/j.diagmicrobio.2014.02.002.

[73] McElvania TeKippe E, Burnham C-AD. Evaluation of the Bruker Biotyper and VITEK MS MALDI-TOF MS systems for the identification of unusual and/or difficult-to-identify microorganisms isolated from clinical specimens. Eur J Clin Microbiol Infect Dis 2014;33:2163–71. https://doi.org/10.1007/s10096-014-2183-y.

[74] Angeletti S, Dicuonzo G, Avola A, Crea F, Dedej E, Vailati F, et al. Viridans Group Streptococci Clinical Isolates: MALDI-TOF Mass Spectrometry versus Gene Sequence-Based Identification. PLoS One 2015;10:e0120502. https://doi.org/10.1371/journal.pone.0120502.

[75] Méndez-Sotelo BJ, Delgado-Beltrán M, Hernández-Durán M, Colín-Castro CA,

Esquivel-Bautista J, Ortega-Oliva SA, et al. In vitro activity of ceftazidime/avibactam, cefiderocol, meropenem/vaborbactam and imipenem/relebactam against clinical strains of the Stenotrophomonas maltophilia complex. PLoS One 2024;19:e0298577. https://doi.org/10.1371/journal.pone.0298577.

[76] Patel J. 16S rRNA gene sequencing for bacterial pathogen identification in the clinical laboratory. Mol Diagnosis 2001;6:313–21. https://doi.org/10.1054/modi.2001.29158.

[77] Drancourt M, Bollet C, Carlioz A, Martelin R, Gayral J-P, Raoult D. 16S Ribosomal DNA Sequence Analysis of a Large Collection of Environmental and Clinical Unidentifiable Bacterial Isolates. J Clin Microbiol 2000;38:3623–30. https://doi.org/10.1128/JCM.38.10.3623-3630.2000.

[78] Sturt AS, Yang L, Sandhu K, Pei Z, Cassai N, Blaser MJ. Streptococcus gallolyticus Subspecies pasteurianus (Biotype II/2), a Newly Reported Cause of Adult Meningitis. J Clin Microbiol 2010;48:2247–9. https://doi.org/10.1128/JCM.00081-10.

[79] Church DL, Cerutti L, Gürtler A, Griener T, Zelazny A, Emler S. Performance and Application of 16S rRNA Gene Cycle Sequencing for Routine Identification of Bacteria in the Clinical Microbiology Laboratory. Clin Microbiol Rev 2020;33. https://doi.org/10.1128/CMR.00053-19.

[80] Vashisht V, Vashisht A, Mondal AK, Farmaha J, Alptekin A, Singh H, et al. Genomics for Emerging Pathogen Identification and Monitoring: Prospects and Obstacles. BioMedInformatics 2023;3:1145–77. https://doi.org/10.3390/biomedinformatics3040069.

[81] Richter M, Rosselló-Móra R. Shifting the genomic gold standard for the prokaryotic species definition. Proc Natl Acad Sci 2009;106:19126–31.

https://doi.org/10.1073/pnas.0906412106.

[82] Ming D, Chen Q, Chen X. Analysis of resistance genes in pan-resistant Myroides odoratimimus clinical strain PR63039 using whole genome sequencing. Microb Pathog 2017;112:164–70. https://doi.org/10.1016/j.micpath.2017.09.012.

[83] Yartasi E, Durmaz R, Ari O, Mumcuoglu I, Dinc B. Molecular characterization of the multi-drug resistant Myroides odoratimimus isolates: a whole genome sequence-based study to confirm carbapenem resistance. Int Microbiol 2023. https://doi.org/10.1007/s10123-023-00472-9.

[84] Liu D, Zhai W, Song H, Fu Y, Schwarz S, He T, et al. Identification of the novel tigecycline resistance gene tet(X6) and its variants in Myroides, Acinetobacter and Proteus of food animal origin. J Antimicrob Chemother 2020;75:1428–31. https://doi.org/10.1093/jac/dkaa037.

[85] Pompilio A, Galardi G, Verginelli F, Muzzi M, Di Giulio A, Di Bonaventura G. Myroides odoratimimus Forms Structurally Complex and Inherently Antibiotic-Resistant Biofilm in a Wound-Like in vitro Model. Front Microbiol 2017;8. https://doi.org/10.3389/fmicb.2017.02591.

[86] Xu S, Chen Y, Fu Z, Li Y, Shi G, Xu X, et al. New Subclass B1 Metallo-β-Lactamase Gene from a Clinical Pathogenic Myroides odoratus Strain. Microb Drug Resist 2018;24:909–14. https://doi.org/10.1089/mdr.2017.0233.

[87] Mammeri H, Bellais S, Nordmann P. Chromosome-Encoded β-Lactamases TUS-1 and MUS-1 from Myroides odoratus and Myroides odoratimimus (Formerly Flavobacterium odoratum), New Members of the Lineage of Molecular Subclass B1 Metalloenzymes. Antimicrob Agents Chemother 2002;46:3561–7. https://doi.org/10.1128/AAC.46.11.3561-3567.2002.

[88] Qin S, Xiao W, Zhou C, Pu Q, Deng X, Lan L, et al. Pseudomonas aeruginosa: pathogenesis, virulence factors, antibiotic resistance, interaction with host, technology advances and emerging therapeutics. Signal Transduct Target Ther 2022;7:199. https://doi.org/10.1038/s41392-022-01056-1.

[89] Kurz CL. Virulence factors of the human opportunistic pathogen Serratia marcescens identified by in vivo screening. EMBO J 2003;22:1451–60. https://doi.org/10.1093/emboj/cdg159.

[90] Gonzalez-Perez MN, Murcia MI, Parra-Lopez C, Blom J, Tauch A. Deciphering the virulence factors of the opportunistic pathogen Mycobacterium colombiense. New Microbes New Infect 2016;14:98–105. https://doi.org/10.1016/j.nmni.2016.09.007.

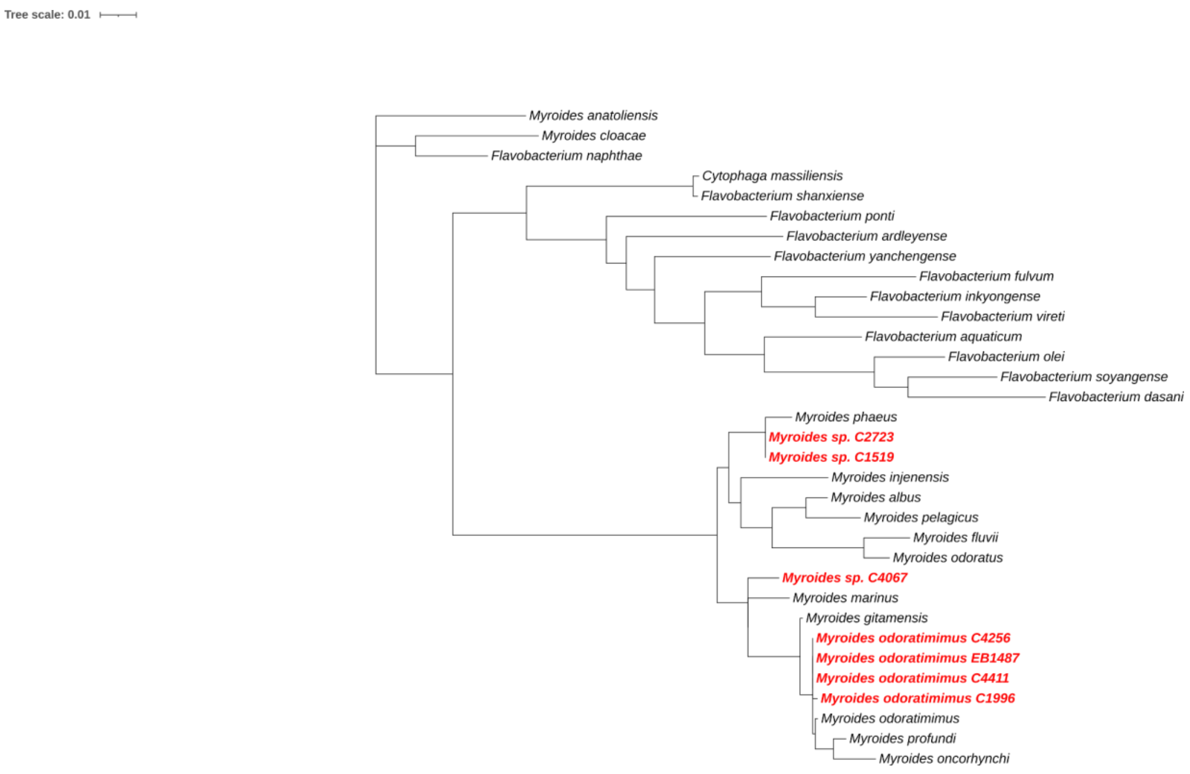

Figure 1. ML phylogenetic tree of the 16S rRNA sequences. The strains evaluated in this study are marked in bold blue color into the tree. Numbers on the nodes and branches represent the Ultrafast Bootstrap values of 10,000 replicates. Scale bar represents the number of nucleotide differences between branches.

Figure 2

Click here to access/download;Figure;Figure 2.docx

Figure 2A Virulence Factors identified in clinical strains of *Myroides spp.*

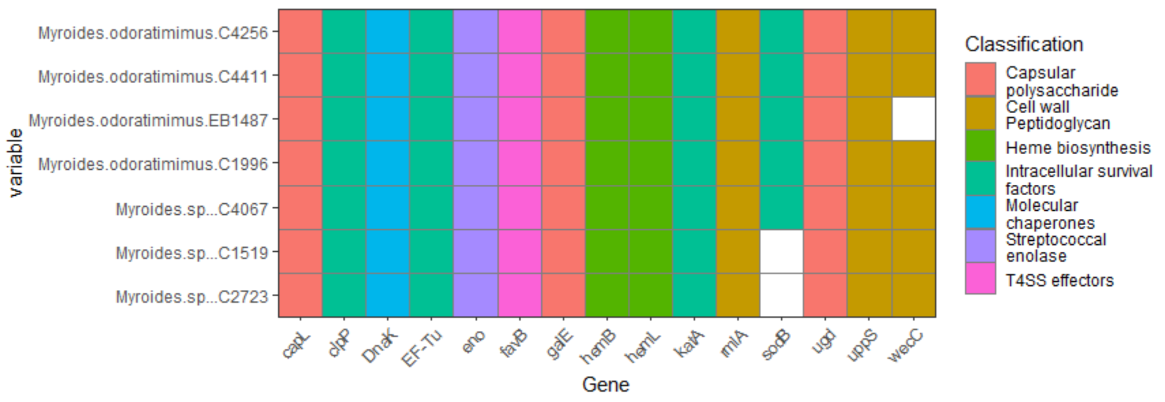

Figure 2B Antimicrobial Resistance Elements presents in *Myroides* species strains.

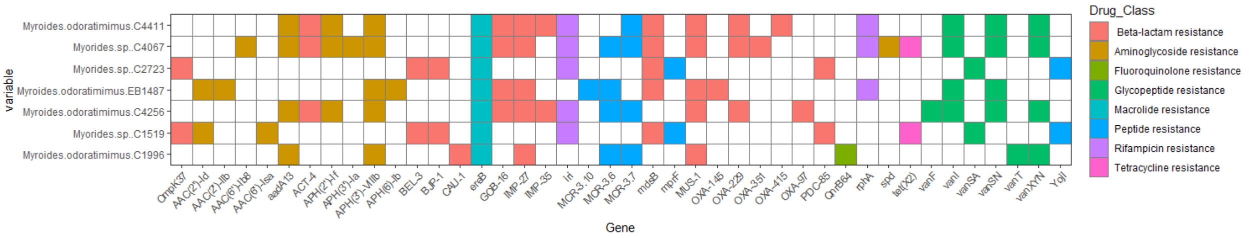

Figure 2. VF (A) and AMR genes (B) predicted in clinical strains of *Myroides odoratimimus* and *Myroides* spp. isolated from Instituto Nacional de Rehabilitación Luis Guillermo Ibarra Ibarra (INR, by its Spanish acronym). The binary map represents the presence/absence of VF in each strain, cataloged by physiological function (classification) in the right legend bar.

Colored boxes represent presence, while white boxes represent absence. The X axis represents the genes, and the Y axis represents the strains (variable).

3(a)

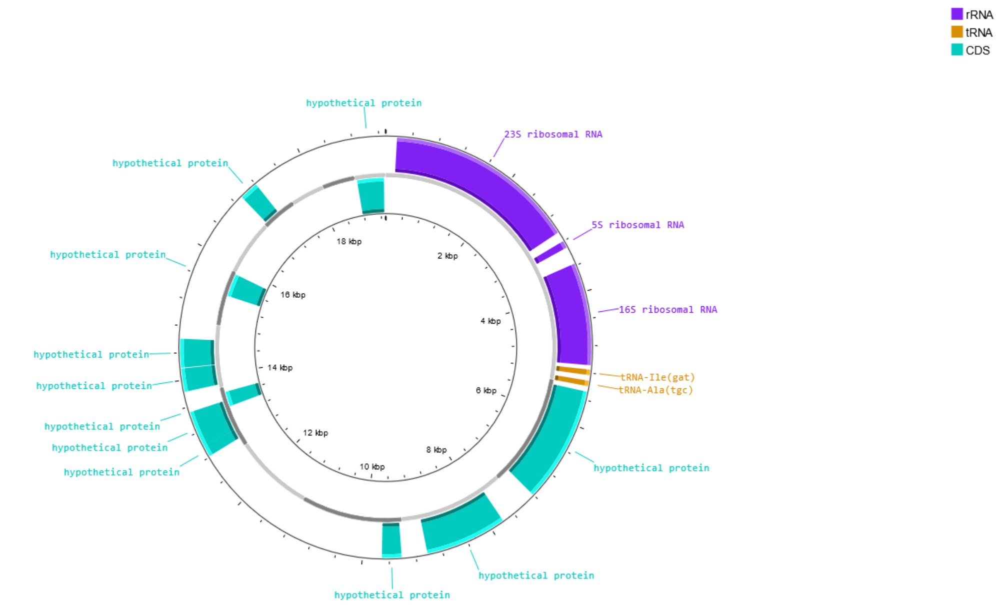

C2723_S6

3(b)

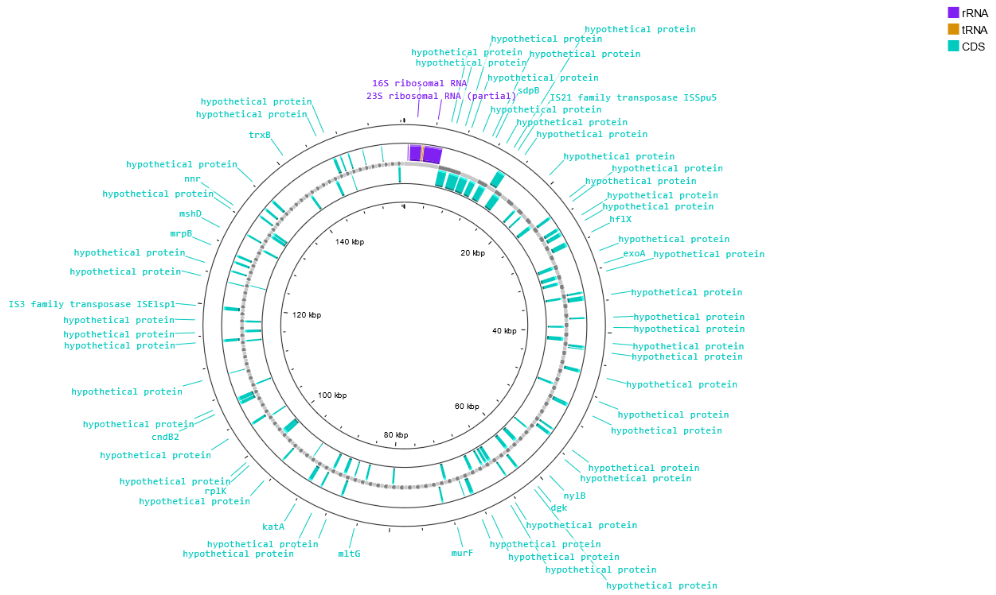

EBL487_S7

Figure. 3. Genomic Map of the identified plasmids pM2723(a) and EB1487_S7 (b). The inner circle represents the genome size per region. The outer circle marks all open reading frames and proteins predicted. Forms and letters in purple indicate the location of ribosomal RNA genes. Forms and letters in orange indicate the location of tRNA genes. Forms and letters in blue indicate the location of annotated and unannotated proteins.

Click here to access/download;Figure;Figure 1S.docx

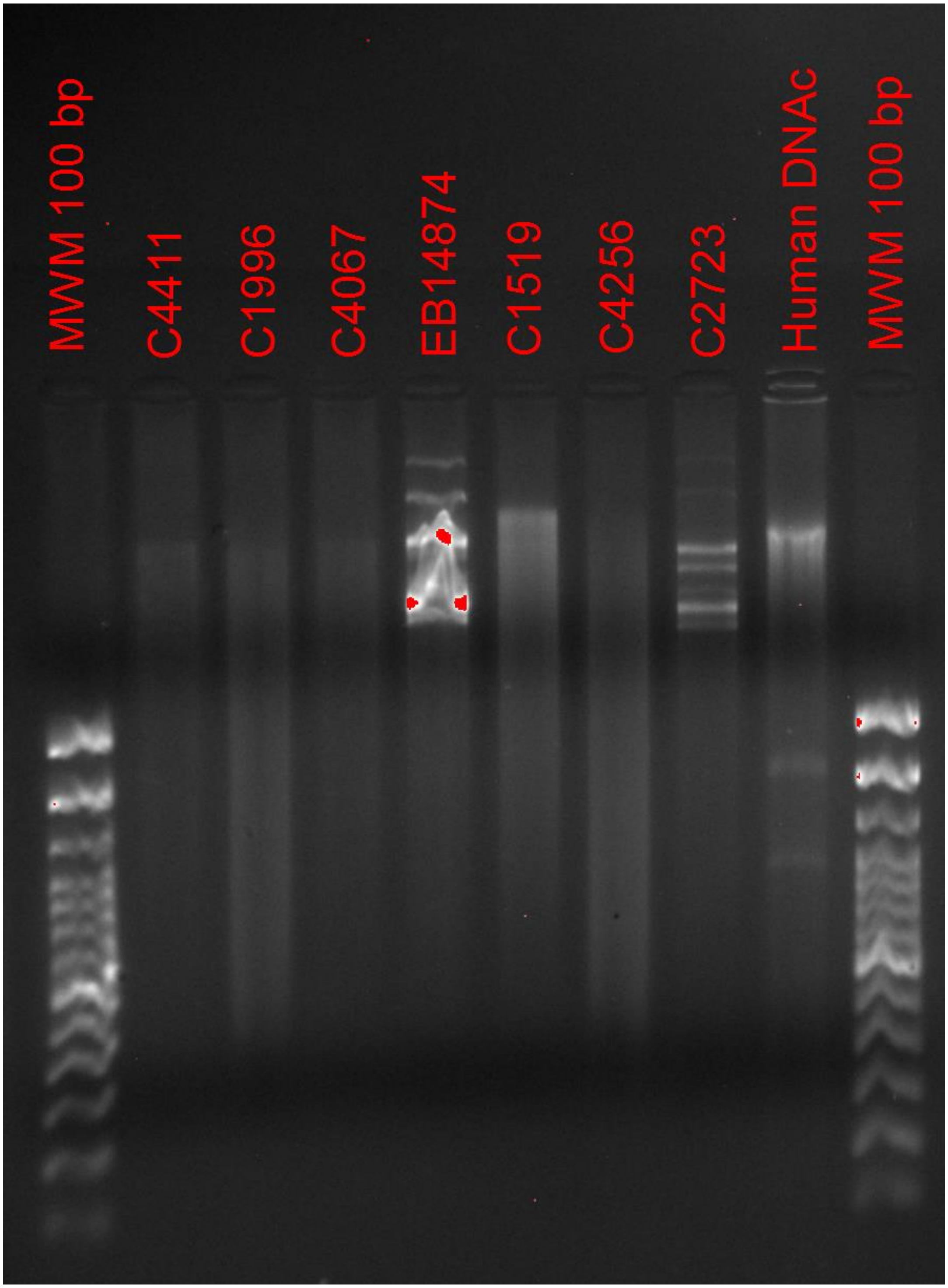

Figure 1S. Plasmids extracted from *Myroides spp.* Clinical strains.

MWM: Molecular weight marker.

Click here to access/download
Supporting Information
55 22 AC_English.pdf
